# Modified mixed nanomicelles with collagen peptides enhanced oral absorption of Cucurbitacin B: preparation and evaluation

**DOI:** 10.1080/10717544.2018.1425773

**Published:** 2018-04-02

**Authors:** Lan Tang, Lulu Fu, Zhuanfeng Zhu, Yan Yang, Boxuan Sun, Weiguang Shan, Zhenhai Zhang

**Affiliations:** a College of Pharmaceutical Science, Zhejiang University of Technology, Hangzhou, PR China;; b Key Laboratory of New Drug Delivery System of Chinese Materia Medica, Jiangsu Provincial Academy of Chinese Medicine, Nanjing, PR China

**Keywords:** Collagen peptides, nanomicelles, Cucurbitacin B, oral administration, transmembrane absorption

## Abstract

Polymer nanoparticles modified with collagen peptides (CPs) are an attractive strategy for the oral delivery of active ingredients from Chinese medicine. Thus, in the present study, collagen cationic CPs were simply separated using ion-exchange resin from bovine CPs, to modify mixed nanomicelles (MMs) on the surface to improve the oral bioavailability of Cucurbitacin B (CuB). The physicochemical property of micelles was characterized, which confirmed the successful modification of the nanomicelles. CPs-modified nanomicelles *in vitro* were found to significantly increase cellular uptake and transportation. Compared to unmodified micelles, the quantity of CPs-modified micelles internalized by Caco-2 cells were 3.74 times greater and the cumulative transportation flux (AP-BL) was 2.81 times greater. The membrane transportation process of CuB-MMs-CPs was found to be associated with energy consumption and clathrin- and caveolin-mediated endocytosis. *In vivo* studies performed on rats indicated that in comparison to CuB and CuB-MMs, the relative bioavailability of CuB-MMs-CPs increased by 3.43 times and 2.14 times, respectively. In addition, the tumor inhibition caused by CuB-MMs-CPs was increased significantly. Therefore, the nanomicelles co-modified with isolated CPs could act as attractive carriers for oral delivery of CuB.

## Introduction

Application of traditional Chinese medicine in the clinical setting prioritizes the route of oral administration, so the efficacy of the administered drug mainly depends on the absorption of active compositions in the intestinal system. However, most of the active ingredients from Chinese medicine usually possess low solubility or low permeability through the intestinal epithelial membrane, and even when associated with efflux pumps, their oral bioavailability is considerably low, which restricts the performance of traditional Chinese medicine in terms of clinical efficacy. Oral delivery of these drugs has remained a great challenge and attracted much attention during recent years (Xie et al., 2011).

A considerable amount of research on topics such as liposomes, micelles, and solid lipid nanoparticles have been reported to improve drug solubility (Gao et al., 2012; Tapeinos et al., 2017; Zhang et al., 2017). Currently, the carrier that has been reported to potentially promote drug transport across the membrane mainly includes plant lectins, membrane fusion proteins, and cell-penetrating peptides (CPPs) (Mora et al., 2016; Zheng et al., 2016; Zhu et al., 2016). Among them, CPPs, a series of short peptides, were found to be supremely effective in enhancing penetration through biological membranes for different cargos, such as micelles, liposomes, and solid lipid nanoparticles (Jiang et al., 2012; Ramsey & Flynn, 2015). The CPPs were usually prepared by solid phase peptide synthesis or prokaryotic expression, the method which was considered complex and expensive.

Collagen peptides (CPs) are a kind low-molecular peptides hydrolyzed from collagen, which mainly composed of gylcine, hydroxyproline, and arginine. They possess short sequences with approximately 2–20 amino acids in length that exert physiological benefits when consumed *in vivo* (Senevirathne & Kim, 2012). After ingestion, the collagen peptides can be absorbed in the intestine and enter the blood stream directly (Erdman et al., 2008). A growing number of literatures demonstrated that peptides from marine organisms had wide spectrum of effects, such as antihypertensive, immunomodulating, anticancer, and antioxidative (Vercruysse et al., 2005; Yang et al., 2010). The collagen peptides (3000 Da, cationic) in our study were simply separated by ion-exchange resin from bovine CP, which could also promote the gastrointestinal absorption of different cargos.

Cucurbitacins are a group of tetracyclic triterpenoid molecules, extracted from *Pedicellus Melo*, that have shown potent anti-proliferative activity against various human cancer cell lines and are used to treat hepatitis and primary liver cancer in the clinical setting (Sliva et al., 2015). Cucurbitacin B (CuB) is one of the most abundantly and widely studied members among cucurbitacins (Chen et al., 2012; Rios et al., 2012). Emerging evidence has shown that CuB is a potential anti-cancer drug for hepatocellular carcinoma, leukemia, non-small cell lung cancer, pancreatic cancer, and breast cancer (Tannin-spitz et al., 2007; Thoennissen et al., 2009; Chan et al., 2010a,b; Kausar et al. 2013). However, previous studies have shown that CuB is classified in the fourth category of best supportive care and thus possesses low solubility and low permeability, which has limited its oral efficacy and clinical application (Zhang et al., 2005; Molavi et al., 2008).

**Figure 1.**
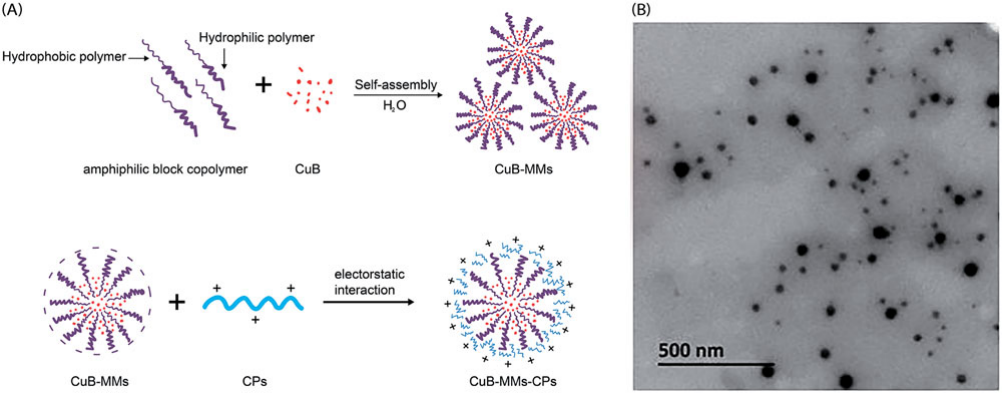
Formation scheme and transmission electron micrographs of CuB-MMs-CPs.

With the goal of overcoming the challenge of CuB’s poor solubility and permeability, the present study aimed to investigate a novel mixed micelles modified by CPs. Mixed micelles system could be used to improve the unfavorable solubility and bioavailability of hydrophobic drugs (Joshi et al., 2016; Zhang et al., 2017). The mixed nanomicelles in the paper composed of Labrasol and Kolliphor HS 15 not only could improve the solubility of CuB, but also negatively charged. Both Labrasol and Kolliphor HS 15 have been used as drug solubilizer and absorption enhancer in many preparation. Labrasol is an amphiphilic nonionic surfactant composed by caprylocaproyl macrogol-8 glyceride, which was used to enhance the membrane permeability (Kenjiro et al., 2006). Kolliphor HS 15 has been reported as an effective P-gp inhibitor, which can alter plasma binding, enhance adsorption, and induce significant effects on the pharmacokinetics (Hou et al., 2016). In the CPs-modified CuB-mixed nanomicelles (CuB-MMs-CPs), the positively charged CPs could clad to the surface of a negatively charged mixed nanomicelle, which when loaded with a drug, would allow efficient transmembrane absorption of the drug (Figure 1(A)). To verify this concept, the basic properties of the CuB micelles were evaluated, their cytotoxicity was explored *in vitro*, and the membrane-penetrating and absorption-enhancing capabilities of the CP-complexed micelles were evaluated using a Caco-2 cell monolayer model *in vitro*. In addition, there *in vivo* pharmacokinetics and oral administration pharmacodynamics were investigated.

## Materials and methods

### Materials and animals

CPs (1000, 3000, 5000 Da) were purchased by Cargill (Minneapolis, MN), and the positively charged CPs were separated from the CPs in our lab. *Pedicellus Melo* was purchased from Aomiao Chinese herbal medicine (Anhui, China) and CuB was extracted and separated from *Pedicellus Melo*. CuB and Cucurbitacin D reference substances were purchased from Weikeqi Biotech Co., Ltd. (Sichuan, China). Labrasol was kindly donated by Gattefosse Trading Co., Ltd (Shanghai, China). Kolliphor HS 15 was generously supplied by Shanghai Yunhong Chemical Preparation Technology Co., Ltd (Shanghai, China). Soybean phospholipid (PC ≥90% of injection class) was purchased from Shanghai Taiwei Pharmaceutical Co., Ltd. (Shanghai, China). Sodium dodecyl sulfate was purchased from Aladdin Reagent Co., Ltd. (Shanghai, China). Corning transwell polycarbonate membrane 24-well plates and 24/96-well plates were purchased from Corning Co., Ltd. (Corning, NY). Hank’s balanced salt solution (HBSS), Dulbecco's modified Eagle's medium (DMEM), phosphate-buffered saline (PBS), essential amino acid, pancreatic enzymes, and penicillin–streptomycin were purchased from Gino Biomedical Technology Co., Ltd. (Zhejiang, China). TransSerum HQ Fetal Borine Serum was obtained from GIBCO Laboratories (Grand Island, NY). Chlorpromazine and verapamil were purchased from Aladdin Reagent Co., Ltd. (Shanghai, China). Sodium azide, indomethacin, and luciferin were obtained from Sigma Co., Ltd. (St. Louis, MO). All other chemicals were of analytical grade or HPLC grade.

Male Sprague-Dawley rats (180–200 g) (certification No. 2014-0001) and nude mice (4–6 weeks old) were obtained from the Experimental Animal Center of Zhejiang province (Zhejiang, China). All animal experiments complied with the law and ethics of the People’s Republic of China on the use of experimental animals.

### Preparation of CuB-loaded mixed micelles

CuB-MMs-CPs were prepared using the film dispersion method. Briefly, CuB and soybean phospholipid (at a ratio of 1:5) were dissolved in 7 mL of methanol solvent, followed by agitation at 40 °C for 45 min until a clear mixture was formed. Next, Labrasol and Kolliphor HS 15 (ratio of 7:3), dissolved in 5 mL of methanol solvent, were applied to the mixture with agitation at 40 °C for another 45 min. Following this, a rotary evaporator was used to evaporate the methanol, and a pressure blowing concentrator was then employed to completely remove the organic solvent, followed by nitrogen blowing for 30 min to form a thin film. Finally, 17 mL of pre-warmed distilled water was administered to the thin film. The thin film was hydrated by using an ultrasonic instrument at 40 °C for 1 h and the CuB mixed nanomicelles (CuB-MMs) were prepared after subjecting them to centrifugation at 10,000 rpm for 20 min. CPs were added to the CuB-MMs solution with agitation at 37 °C for 30 min. The CuB-MMs-CPs were obtained *via* filtration to remove any insoluble substances by using the cellulose acetate filters (pore size: 0.45 μm).

The control micelles were prepared by the same method without addition of CuB at any stage of the preparation.

### Isolation and preparation of positively charged CPs

CP solutions (0.1 g/mL) with molecular weights of 1000 Da, 3000 Da, and 5000 Da were prepared, respectively. The solutions were then mixed with ion-exchanged resin in a 1:1 proportion (w/v). The ion-exchanged resins absorbing CPs were collected after shaking in a swing bed for 3 h at 40 °C. The CPs absorbed in ion-exchanged resin were separated into three fractions by using a step gradient static elution of 0.2 M, 0.5 M, and 1 M NaCl. Fractions of 0.5 M and 1 M NaCl solution were merged for the principal study. Afterwards, the nanofiltration membrane separation and dialysis methods were applied to remove the NaCl in the CPs obtained previously and concentrate the CP solution. Following this, lyophilization was applied to dry the CPs of different molecular weights were prepared in the same manner. The purity of CPs of each molecular weight was determined.

### Optimization and characterization of CPs

CuB-MMs modified with different amounts of CPs (30 mg, 60 mg, 90 mg, 120 mg, 150 mg, and 180 mg) in each molecular weight (1000 Da, 3000 Da, and 5000 Da) were prepared. Their uptake assays in Caco-2 monolayer film were introduced to evaluate the influence of quantity and molecular weight of CPs upon uptake. The cells were treated with CuB-MMs-CPs (CuB 50 μg/mL) and CuB-MMs (CuB 50 μg/mL) for 4 h. In addition, amino acid compositions of the optimal CPs and original CP of the same molecular weight were investigated *via* an automatic amino acid composition analyzer, the isoelectric point (IP) of CPs were determined by acid–base precipitation.

### Characterization of micelles

#### Particle size and zeta potential

The particle size and zeta potential were determined *via* a laser size scattering determinator (DelsaNano C, BECKMAN COULTER, Fullerton, CA). All measurements were performed in triplicate.

#### Morphology observation

The morphology of CuB-MMs-CPs was observed under a transmission electron microscope (TEM, JEM-1200EX, Japan Electronics Corporation, Tokyo, Japan). The sample was diluted 30-fold with distilled water, and some solution was applied on the copper screen for volatilization. Following this, a drop of 2% phosphotungstic acid solution was used for staining. After drying for 20 min, these micelles were put under the TEM for morphology observation.

#### Determination of entrapment efficiency and drug loading

The CuB-MMs-CPs were analyzed for their CuB concentration using HPLC (Agilent 1200, Agilent Technologies, Santa Clara, CA) equipped with a UV detector set at 228 nm. All experiments were performed in triplicate to calculate the encapsulation efficiency (EE) and drug loading (DL) *via* the following equations:

$$EE\%=\frac{Total\;amount\;of\;CuB\;in\;micelles}{Total\;amount\;of\;CuB\;added}\times 100$$

$$DL\%=\frac{Total\;amount\;of\;CuB\;in\;micelles}{Weight\;of\;micelles}\times 100$$

#### *In vitro* CuB release

The *in vitro* release behavior of CuB from CuB-MMs-CPs micelles was monitored in a phosphate buffered saline (PBS) (PH 6.8) medium containing 0.5% sodium dodecyl sulfate (SDS) to obtain pseudo sink conditions. Briefly, aliquots of CuB-MMs-CPs micelles were introduced into a dialysis bag (MWCO =2000 Da), the sealed end of which was immersed fully into 40 mL of a release medium at 37 °C. This medium was stirred at 100 rpm for 24 h. At fixed time intervals, 1 mL aliquots were withdrawn and replaced with an equal volume of fresh medium. CuB release from the stock solution was conducted under the same conditions as the controls. The concentration of CuB in the samples was determined by HPLC.

### Cell studies

#### Cytotoxicity

The *in vitro* cytotoxicity of CuB-MMs-CPs was assessed using the MTT assay. HepG-2 cells were seeded onto 96-well plates in 200 μL. DMEM was applied to obtain a concentration of 5000 cells per well and cultured at 37 °C, 5% CO_2_ for 24 h to allow attachment of cells. The cells were then incubated for 48 h with 200 μL medium containing a series of blank micelles, CuB-MMs-CPs, CuB-MMs, and CuB, of which the concentration of CuB ranged from 0.001 μM to 100 μM. The percentage of cell viability was calculated on the basis of the optical density values at 490 nm of the sample wells, which contained cells treated with the culture medium.

#### Caco-2 cell culture

Human colon carcinoma Caco-2 cells were cultured in 75-cm^2^ culture flasks with culture medium. The DMEM contained 10% fetal bovine serum, 1% non-essential amino acids, 1% L-glutamine, 100 mg/mL streptomycin, and 100 IU/mL penicillin. The culture flasks were maintained in an incubator at 37 °C, 95% relative humidity, with 5% CO_2._ Upon reaching 80% confluence, cells were harvested. They were then detached from the culture flasks by trypsinization with 0.25% trypsin containing 0.05 mM ethylenediamine tetraacetic acid, and diluted to a density of 1.0 × 10^5^ cells/mL. The cell suspensions were seeded onto 24-well plates for the uptake assay. Some cells were diluted to a density of 3 × 10^5^ cells/mL and seeded onto several transwell plates for transcellular permeation study. Caco-2 cells were grown in DMEM as described above for 21 days until their transepithelial electrical resistance (TEER) achieved a constant value (TEER >500 Ω cm^2^). TEER values were measured using a Millicell-ERS (Millipore, MA).

#### Uptake assay

Caco-2 cells were grown on 24-well culture plates in DMEM for 14 days. HBSS pre-warmed at 37 °C was used to rinse the cells twice and then 1 mL HBSS was added into each well and incubated for 30 min and subsequently discarded. All samples were filtered through a 0.22 μm filtering film for sterilization. Subsequently, 1 mL of CuB-MMs-CPs, CuB-MMs, and CuB (CuB 12.5 μg/mL, 25 μg/mL, 50 μg/mL, 100 μg/mL, 200 μg/mL) were added into the wells and discarded after 2 h to evaluate the uptake promotion of CPs. Some cells were treated with CuB-MMs-CPs, CuB-MMs, and CuB (CuB 50 μg/mL) for defined intervals (0.5 h, 1 h, 1.5 h, 2 h) to study the effect of the time of CuB on uptake. To assess the effect of temperature on uptake, a group of cells were incubated with the micelles at 4 °C for 2 h. After co-incubation with all the samples, the cells were washed at least thrice with cold HBSS to terminate cellular uptake and eliminate the surface-bound micelles. In terms of qualitative analysis, 0.1% lauryl sodium sulfate was added to stimulate cell lysis. Upon undergoing transfer by methanol and collection by centrifugation at 10,000 rpm for 5 min, the supernatant was obtained and analyzed by HPLC.

#### Transcellular transport study

Caco-2 cells were grown in 24-well transwell culture plates in DMEM for 3 weeks. The intactness of the tight junction barrier of the Caco-2 cell monolayer was monitored based on their trans-epithelial electrical resistance (TEER). First, the culture medium in the apical (AP) and basolateral (BL) chambers was replaced with HBSS, and the plate was incubated for 30 min at 37 °C. Then, the Caco-2 cells were rinsed twice with HBSS, and CuB-MMs-CPs (at concentrations of CuB were 25 μg/mL, 50 μg/mL, 100 μg/mL, and 200 μg/mL) were dispersed in 0.3 mL HBSS on the apical side, and 1.2 mL HBSS was applied to the basolateral side. The plates were incubated at 37 °C. At the determined time points (0.5 h, 1 h, 1.5 h, and 2 h), 0.6 mL of the samples was removed from the basolateral chamber and an equal volume of HBSS was supplemented. After the micelles were destroyed by the addition of methanol, the amount of transported CuB was determined using HPLC and calculated *via* the standard curve for CuB. In addition, the effect of temperature (4 °C and 37 °C) and inhibitors (sodium azide, indomethacin, chlorpromazine, and verapamil) was also investigated, and treatments were as described above. All experiments were performed in triplicate. Cellular transportation from the basolateral side to the apical side was also investigated in each condition.

### Evaluation of pharmacokinetics

Sprague-Dawley rats were divided randomly into three groups with six in each group and fasted overnight with free access to a water supply prior to experimentation. The test samples including CuB, CuB-MMs, and CuB-MMs-CPs were orally administered to each group at a CuB dose of 1 mg/kg. Blood samples were taken from the retro-orbital plexus of rats prior to drug administration at defined time points after dosing, and centrifuged immediately at 8000 rpm for 5 min. Following this, 300 μL of plasma sample was added with 800 μL ethyl acetate, and the mixture was thoroughly vortexed and centrifuged at 8000 rpm for 10 min. The supernatant was analyzed using HPLC.

### Pharmacodynamic evaluation

For *in vivo* study, we used a nude mouse as a model for HepG-2 breast cancer. When the tumor reached approximately 100–150 mm^3^, mice were randomly assigned to three groups (*n* = 8 animals per group) treated with one of the following: normal saline, control CuB-MMs, and CuB-MMs-CPs (equivalent CuB dose, 2 mg/kg). All treatments were orally administered on days 1, 3, 5, 7, 9, 11, 13, 15, 17, 19, and 21. The volume of the tumor was measured daily. On day 22, mice were killed and tumors were weighed.

## Results

### Optimization and characterization of CPs

The purities of 1000 Da, 3000 Da, 5000 Da CPs purified are 91.36 ± 2.62%, 96.07 ± 3.33%, and 93.28 ± 2.57% respectively. All the purities of CPs isolated were greater than 90%.

**Figure 2.**
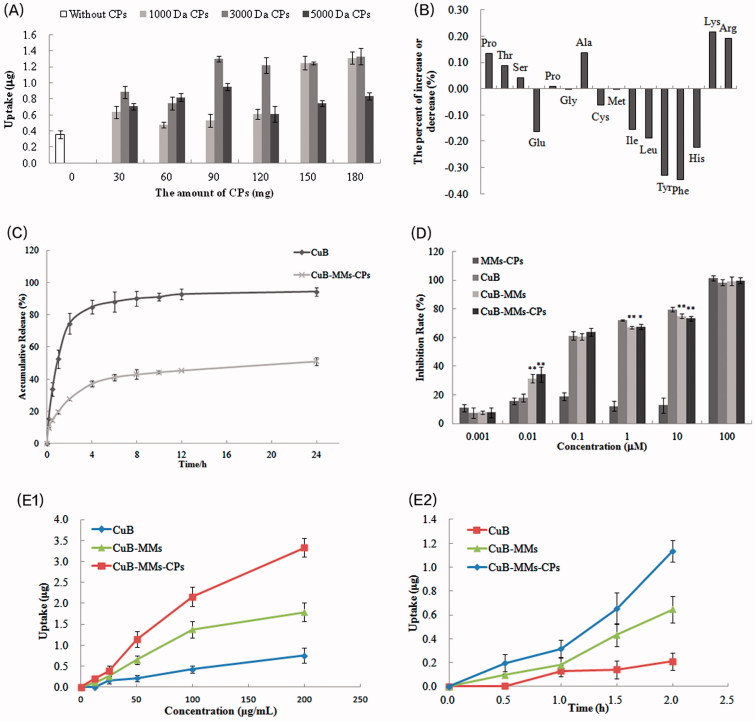
Optimization of CPs, cytotoxicity and uptake evaluation of CuB and CuB-MMs-CPs. The effects of CPs’ different molecular weight and quantity on Caco-2 intaking of CuB (A). Changes of amino acid ratio between before and after the separation of CPs (B). CuB release profiles from the micelles *in vitro* (C). Cytotoxicity of blank MMs-CPs, CuB, CuB-MMs, and CuB-MMs-CPs against HepG -2 cells (D, *n* = 3). Cellular uptake efficiency of the CuB, CuB-MMs, and CuB-MMs-CPs micelles by Caco-2 cells in different concentrations (12.5 μg/mL, 25 μg/mL, 50 μg/mL, 100 μg/mL, 200 μg/mL) or after 0.5 h, 1 h, 1.5 h, and 2 h incubation (E).

The results presented in Figure 2(A) showed that owing to being wrapped by CPs, CuB-MMs-CPs were internalized by Caco-2 cells in a more efficient manner, in comparison to CuB-MMs. With CPs modification, the maximum quantity of CuB internalized by Caco-2 cell was 3.74 times higher at 4 h than that of unmodified micelles. Among them, the intake of CuB increased overall upon an increase of 1000 Da and 3000 Da CPs. In addition, the intake of micelles showed little difference at 5000 Da CPs with different addition. Although 90 mg of 3000 Da CPs-complexed micelles had almost the same intake as 150 mg of 1000 Da CPs, considering the amount of carrier material and comparative drug loadings, 3000 Da CPs were chosen as the transmembrane material at a dosage of 90 mg.

The changes in amino acid compositions between 3000 Da original CP and 3000 Da positively charged CPs are listed in Figure 2(B). Experimental results indicated that the content of the amino acids Hyp, Thr, Ser, Pro, Ala, Lys, and Arg significantly increased. Simultaneously, the contents of Glu, Cys, Ile, Leu, Tyr, Phe, and His were correspondingly decreased. Precisely, the basic amino acid Lys and Arg increased while acidic amino acid decreased, which supposed the alkaline CPs might obtained from the separation process. In addition, the 3000 Da CPs’ isoelectric point might be between 6.82 and 7.33 as it was a mixture peptides, and it also indicated that the CPs would be positively charged in physiological conditions basically.

### Characterization of nanomicelles

The transmission electron micrographs of CuB-MMs-CPs-complexed micelles are displayed in Figure 1(B). As shown, CuB-MMs-CPs-complexed micelles were generally spherical with a uniformly sized distribution. The characteristics of the micelles are summarized in Table 1. The results demonstrated that CuB-MMs, with or without CP modification were of different particle sizes, and the size of CuB-MMs-CPs were considerably larger than that of the CuB-MMs. In addition, their zeta potential exhibited a significant change from –23.06 mV to +3.62 mV after the CPs were decorated on their surface. CPs are positively charged under normal physiological circumstances, and therefore, the conversion of the zeta potential from negative to positive was indirect evidence that CPs could be present on the surface of the micelles. Encapsulation efficiencies of these micelles were above 89%, drug loadings were above 5.8%. However, the original CP could not lead to the size and zeta potential change of micelles, as it could not clad to the surface of CuB-MMs, which might be ascribed to it was a mixture of acid and alkali peptides. The separation procedure of original CP lead to the changes in amino composition made the CPs positively charged.

**Table 1.** Particle size and potential of CuB-MMs-CPs-complexed micelles with different amount of CPs (*n* = 3).

	Mean ± SD	
The amount of CPs/mg	Particle size (nm)	Zeta potential (mV)
0	41.8 ± 3.6	−23.06 ± 1.34
30	35.2 ± 2.8	−14.28 ± 0.98
60	59.6 ± 3.6	−6.87 ± 1.64
90	76.5 ± 2.7	3.62 ± 0.37
120	81.3 ± 1.8	3.37 ± 0.67
150	96.8 ± 3.3	3.07 ± 0.64
180	107 ± 2.93	3.53 ± 0.43

The accumulated percentage releases of CuB from the micelles and its stock solution are shown in Figure 2(C). CuB showed an initial burst release in 4 h. CuB-MMs-CPs showed controlled release for 1 day without any burst release. After 24 h of dialysis, the percentage of CuB from mixed micelles was (50.98 ± 2.3)%. Compared to CuB, the micelles showed a significant (*p* < .01) sustained release in the in the *in vitro* drug release.

### Cytotoxicity evaluations

The *in vitro* cytotoxicities of the blank micelles, CuB-MMs-CPs, and CuB are illustrated in Figure 2(D). The MTT assay demonstrated a significant killing effect on HepG-2 after 48 h treatment with CuB-MMs-CPs. Although free CuB also showed a noticeable cytotoxic effect, CuB-MMs-CPs provoked a more significant cell death rate at 0.01 µM of CuB concentrations. Higher dose- and time-dependent cell-killing efficacies of HepG-2 cells by CuB-MMs-CPs were also observed. For the HepG-2 hepatoma cell line, the IC_50_ value was (0.1039 ± 0.0503) μM for free CuB, whereas the IC_50_ was (0.09239 ± 0.0383) μM for CuB-MMs and (0.07695 ± 0.0442) μM for CuB-MMs-CPs. Thus, our results demonstrated that HepG-2 hepatoma cells were more sensitive and had a superior response to CuB-MMs-CPs, compared to those of free CuB.

### Uptake of CuB, CuB-MMs, and CuB-MMs-CPs by Caco-2 cell

The results in Figure 2(E) show that both CUB-MMs and CuB-MMs-CPs were internalized by Caco-2 cells more efficiently compared to free CuB. With CPs modification, the quantity of micelles internalized by Caco-2 cell was 3.41 times higher at 2 h than that of free CuB, 0.87 times higher than that of CuB-MMs at 200 μg/mL concentration of CuB. A smaller difference was found between them at low concentrations and short treatment time. However, the cellular uptake amount of free CuB-MMs and CuB-MMs-CPs increased nonlinearly with increasing concentrations. Temperature significantly affected the cellular uptake of both free CuB and CuB-MMs-CPs. Compared with uptake at 4 °C, the quantity of cellular uptake at incubation at 37 °C was 1.92 times higher for CuB-MMs-CPs, demonstrating that the uptake of micelles by Caco-2 cells was an energy-dependent process.

### Transcellular transportation of CuB-MMs-CPs across the Caco-2 cell monolayer

**Figure 3.**
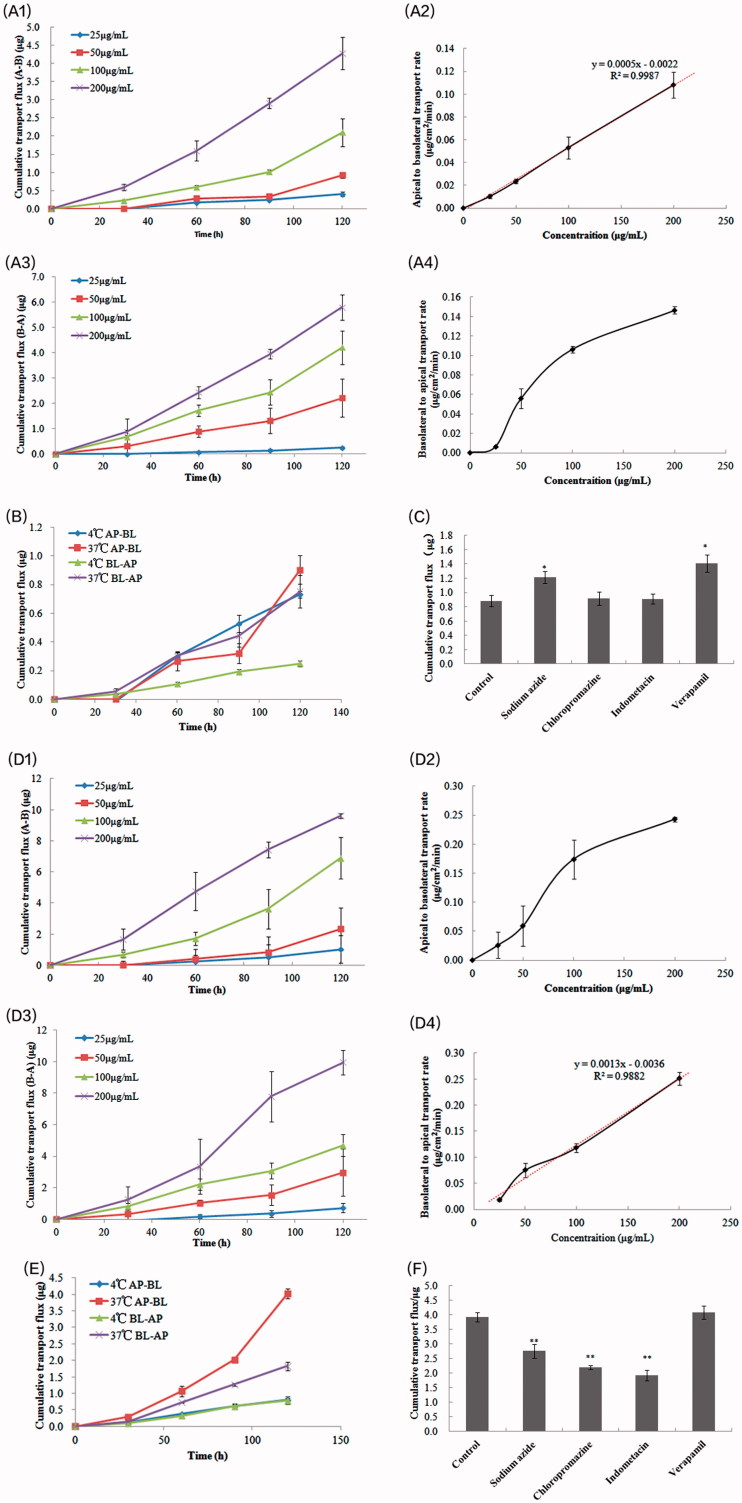
Transcellular transportation evaluation of CuB and CuB-MMs-CPs across the Caco-2 cell monolayer. The cumulative transport flux and transport rate of CuB and CuB-MMs-CPs from AP to BL and BL to AP (A and D). Effects of temperature on the transportation quantity of CuB and CuB-MMs-CPs across Caco-2 cell monolayers from AP to BL and BL to AP were investigated (B and E). The effect of efflux pump, ATP, and endocytosis inhibitors on the transportation of CuB and CuB-MMS-CPs were assayed (C and F).

As illustrated in Figure 3(A), the cellular transportation rate of CuB from AP-BL had linear relation with its concentration. However, the cellular transportation rate of CuB from BL-AP saturated as the concentration of CuB increased. There was no significant effect of temperature on the quantity of cellular transportation of CuB from the AP side to BL side, whereas the cellular transportation quantity of CuB from the BL side to AP side significantly reduced at 4 °C (Figure 3(B)). The effects of various inhibitors are shown in Figure 3(C). After joining the efflux inhibitor verapamil and the ATP inhibitor sodium azide, the Caco-2 cell transport capacity of CuB increased significantly, whereas the endocytosis inhibitors chlorpromazine and indomethacin showed no significant effect on their transportation.

The results presented in Figure 3(D) show that the cellular transportation rate of CuB-MMs-CPs from AP-BL exhibited a nonlinear relation with the concentration of CuB. When the concentration of CuB increased, the increase in the cellular transportation rate showed no apparent trend. However, the cellular transportation rate of CuB-MMs-CPs from BL-AP showed an approximately linear relation. Significant effects of temperature on the quantity of cellular transportation of CuB-MMs-CPs for both the AP-BL and BL-AP directions were observed and the cellular transportation quantity of CuB-MMs-CPs reduced in both transfer directions at 4 °C (Figure 3(E)). The effects of various inhibitors are shown in Figure 3(F). After association with verapamil, the Caco-2 cells’ transport capacity of CuB showed no significant change, whereas the capacities of sodium azide, chlorpromazine, and indomethacin enhanced significantly.

Generally, the cumulative transportation flux (AP-BL) of CPs-modified micelles was 2.25 times higher at 2 h than that of the unmodified nanoparticle at a high concentration of 200 μg/mL, and 2.81 times higher at a low concentration of 25 μg/mL. Therefore, modification of CPs resulted in a significant improvement of transportation across the Caco-2 cell monolayer. The Papp value of CuB-MMs-CPs was 20.22 × 10^–6 ^cm/s in 2 h and increased to 2.25 times compared with unmodified CuB.

### Pharmacokinetics studies

**Figure 4.**
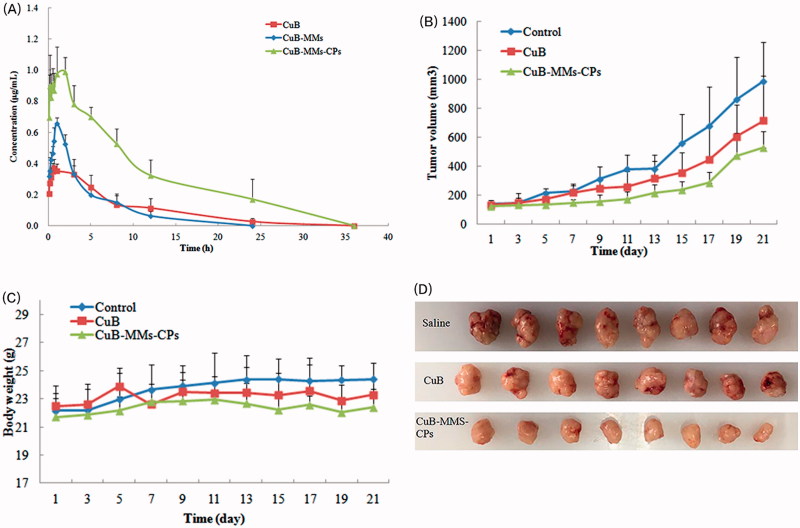
Time–concentration curve for CuD after oral administration of Cucurbitacin B, CuB-MMs, and CuBMMs-CPs (A) and *in vivo* antitumor study of normal saline, CuB, and CuB-MMs-CPs in Balb/c nude mice implanted with HepG-2 cells. Tumor volumes (B) and body weight (C) were monitored. Tumor weight was monitored at the end of the experiment (D). The result was presented as the mean ± SD (*n* = 6).

The pharmacokinetics parameters are summarized in Table 2 and Figure 4(A). The CuB was found to be metabolized as CuD in plasma *via* analyzes and the metabolites in plasma were identified by LC–MS and NMR. The relative bioavailabilities of CuB for CuB-MMs-CPs were 3.43 times and 2.14 times greater than that of free CuB and CuB-MMs, respectively.

**Table 2.** Pharmacokinetic parameters of CuD after oral administration of CuB, CuB-MMs, and CuB-MMs-CPs (*n* = 6).

Parameter	CuB	CuB-MMs	CuB-MMs-CPs
AUC_(0-_*_t_*_)_/µg h mL^−1^	3.50 ± 0.77	5.29 ± 0.69	10.69 ± 1.66
AUC_(0-∞)_/µg h mL^−1^	3.73 ± 0.97	6.00 ± 1.32	12.81 ± 3.73
MRT/h	6.69 ± 1.36	6.99 ± 0.88	7.77 ± 1.82
C_last/µg mL^−1^	0.024 ± 0.021	0.052 ± 0.035	0.135 ± 0.035
*t*_1/2_/h	5.09 ± 2.20	7.71 ± 3.66	7.71 ± 0.11
*T*_max_/h	1.08 ± 0.95	1.00 ± 0.71	1.514 ± 0.403
Vz/*F*/L kg^−1^	1865.20 ± 441.12	1794.30 ± 591.40	833.08 ± 321.71
CLz/*F*/L h^−1^ kg^−1^	286.22 ± 83.85	172.43 ± 32.43	83.84 ± 24.45
*C*_max_/µg mL^−1^	0.40 ± 0.04	0.659 ± 0.036	1.10 ± 0.12

### Pharmacodynamic studies

Orally administered mice bearing HepG-2 xenografts with free CuB showed slightly slower growth than animals administered with saline. This result reflects the poor efficacy of CuB for treating liver tumors. In contrast to free CuB, CuB-MMs-CP-complexed micelles efficiently inhibited tumor growth (Figure 4(B) and (C)). At the end of the 21-day treatment period, the tumor weights of various groups relative to the weight of saline-treated animals were as follows: free CuB 68.54%; CuB-MMs-CP-complexed micelles 37.71%. These results suggest that CuB has significant antitumor efficacy (*p* < .01) when delivered as CuB-MMs-CPs micelles than as a free drug in solution.

## Discussion

Over the recent years, numerous efforts have been underway to improve the oral bioavailability and transmembrane transportation of bioactive components to improve delivery systems. CPPs, such as Tat, R8, and PEP-1 were designed to increase the delivery of bioactive components as drug carriers. For example, modified nanoparticles with Tat and amphipathic chitosan derivatives were used as carriers to improve colonic absorption of the drug (Guo et al., 2017). And R8, Tat, and a secretion peptide (Sec) with an N-terminal stearylation were introduced to modify nanoparticles on the surface to improve the oral bioavailability of peptide and protein drugs (Kaplan et al., 2005; Torchilin, 2008; Milletti, 2012; Liu et al., 2013; Wang et al., 2014). In order to identify natural peptides obtained through convenient and economical techniques rather than complicated and expensive methods, the positively charged CPs were prepared and used in the present study. As demonstrated in the assay, surface modification by the CPs endowed mixed nanomicelles with enhanced cellular internalization and intestinal permeability, both of which improved the oral bioavailability of CuB.

In the evaluation of micelle characteristics, the zeta potential alteration from a negative to positive charge was observed after the CuB-MMs were modified with the CPs. In the meantime, the particle size of the CPs-modified micelles was enhanced accordingly, which indirectly indicated that the CPs were present on the surface of the micelles. However, the positive zeta potential of CuB-MMs-CPs no longer increased, no matter how much the content of CPs increased. The low positive zeta potential may be attributed to the low purity of positive charged CPs. Therefore, the optimization of the CPs preparation technology should be developed in a follow-up study. In the present study, we prepared only three positively charged CPs of different molecular weights. In future studies, further CPs of different molecular weights from different animal skins could be investigated.

CPs have been widely applied as useful carriers for the intracellular delivery of bioactive components, but the exact mechanisms underlying their cellular uptake and transmembrane transportation, especially when they are conjugated with nanoparticles, remain controversial (Brooks et al., 2010; Mager et al., 2010; Bolhassani, 2011; Khafagy & Morishita, 2012). Through *in vitro* cellular uptake studies, the cellular intake of micelles modified with CPs was found to be significantly increased, especially at high concentrations. The rise in their cellular uptake may be due to the electropositivity of CuB-MMs-CP complexed micelles, which overcame the limitations caused by the electrostatic characteristics of the cytomembrane and influenced the membrane permeability. Otherwise, the P-gp inhibition by carrier materials, Labrasol and Kolliphor, also contributed to the cellular intake (Dubray et al., 2016; Hou et al., 2016). If the cellular intake content increased with the concentration of treated compound in nonlinear fashion and became saturated when the concentration achieved a certain thickness, then the procedure could be considered to be mediated with a specific protein carrier on the cell membrane. Therefore, the results of the cellular intake studies suggest that the factor of transporters should also be considered. Alternatively, the results of cellular uptake studies at different temperatures showed that the internalization of CP-modified CuB-MMs by Caco-2 cells occurred *via* an energy-dependent transport pathway.

The transport content has a linear relationship with the concentration and treatment time of the drug, whereas the carrier-mediated transport pathway possesses a non-linear relationship with these factors, and the transport quantity saturates at a given concentration. Therefore, the results from our study of the transmembrane transport behavior and absorption mechanism showed that the transport rate, apparent transport capacity, and apparent permeability coefficient of CuB-MMs-CPs nanomicelles were increased. Its absorption mechanism was also changed, in comparison to that of free CuB. The absorption of CuB was dominated by passive spreading and this occurred in a concentration-dependent manner. Due to the presence of the P-gp efflux effect, the P-gp inhibitor could significantly improve its absorption. The secretory process involved vector-mediated protein involvement, which required energy consumption and might be associated with interstitial protein mediation. The absorption of Cu-MMs-CPs nanomicelles was dominated by active transport, of which the carriers Labrasol and Korlliphor could effectively inhibit the P-gp efflux. The transport process required energy consumption, and its endocytotic proteins might be clathrin and caveolin. The secretion process was mainly controlled by changes in concentration.

The superiority of modification by CPs was subsequently confirmed by *in vivo* pharmacokinetic and pharmacodynamic studies. Compared to the CuB-MMs and free CuB, the relative bioavailabilities and tumor inhibition rate were substantially enhanced by CP modification.

## Conclusion

Our present study was the first investigation to indicate that modified micelles with CPs as carriers could enhance absorption of orally administered CuB. Cellular uptake and transcellular transportation performance of CuB-MMs-CPs were proven to be better than those of CuB-MMs and CuB. In addition, we demonstrated that CuB-MMs-CPs produced the most significant antitumor effect after being ingested into mice bearing HepG-2 xenografts, which suggests their improved penetration efficiency attributed to CPs. Hence, we believe that the use of CPs as carriers may provide an effective method for the oral delivery of drugs, which could potentially overcome the barriers against the drug diffusion and penetration in the intestine.
